# Quantum mechanical static dipole polarizabilities in the QM7b and AlphaML showcase databases

**DOI:** 10.1038/s41597-019-0157-8

**Published:** 2019-08-19

**Authors:** Yang Yang, Ka Un Lao, David M. Wilkins, Andrea Grisafi, Michele Ceriotti, Robert A. DiStasio

**Affiliations:** 1 Department of Chemistry and Chemical Biology, Cornell University, Ithaca, NY 14853 USA; 2 Laboratory of Computational Science and Modeling, IMX, École Polytechnique Fédérale de Lausanne, 1015 Lausanne, Switzerland

**Keywords:** Quantum chemistry, Density functional theory, Chemical physics, Chemical physics, Electronic structure of atoms and molecules

## Abstract

While density functional theory (DFT) is often an accurate and efficient methodology for evaluating molecular properties such as energies and multipole moments, this approach often yields larger errors for response properties such as the dipole polarizability (*α*), which describes the tendency of a molecule to form an induced dipole moment in the presence of an electric field. In this work, we provide static *α* tensors (and other molecular properties such as total energy components, dipole and quadrupole moments, etc.) computed using quantum chemical (QC) and DFT methodologies for all 7,211 molecules in the QM7b database. We also provide the same quantities for the 52 molecules in the AlphaML showcase database, which includes the DNA/RNA nucleobases, uncharged amino acids, several open-chain and cyclic carbohydrates, five popular pharmaceutical molecules, and 23 isomers of C_8_H_n_. All QC calculations were performed using linear-response coupled-cluster theory including single and double excitations (LR-CCSD), a sophisticated approach for electron correlation, and the d-aug-cc-pVDZ basis set to mitigate basis set incompleteness error. DFT calculations employed the B3LYP and SCAN0 hybrid functionals, in conjunction with d-aug-cc-pVDZ (B3LYP and SCAN0) and d-aug-cc-pVTZ (B3LYP).

## Abstract (summary)

**Table:** 

Design Type(s)	chemical structure classification objective • chemical reaction data analysis objective • modeling and simulation objective
Measurement Type(s)	chemical structure analysis
Technology Type(s)	*ab initio* quantum chemistry computational method
Factor Type(s)	atom
Sample Characteristic(s)

Machine-accessible metadata file describing the reported data (ISA-Tab format)

## Background & Summary

The molecular dipole polarizability, ***α***, describes the tendency of a molecule to form an induced dipole moment in the presence of an external electric field. Knowledge of this fundamental response property is central to describing non-bonded interactions (such as induction and dispersion) between molecules in clusters or the condensed phase^1–3^, computing Raman and sum frequency generation (SFG) spectra^4–7^, and developing polarizable force fields^8–12^. When compared to other ground-state molecular properties (*e*.*g*., multipole moments), the theoretical prediction of the ***α*** tensor is considerably more difficult to obtain, as this quantity is often more sensitive to the description of the underlying molecular electronic structure. In this regard, benchmark *ab initio* calculations of ***α*** are quite challenging to perform, as they require a simultaneous treatment of sophisticated electron correlation effects as well as mitigation of basis set incompleteness error to ensure sufficiently accurate and converged results.

To obtain benchmark values for ***α*** in molecular systems with a sizeable HOMO-LUMO gap (*i*.*e*., systems that are well-described by a single-reference wavefunction), one can utilize quantum chemical methods such as linear-response coupled-cluster theory (LR-CC)^13–15^, which provides an accurate and reliable treatment of electron correlation. The downside of such wavefunction-based approaches is the large (and often prohibitive) computational cost associated with the inclusion of higher order excitations in the CC expansion. For example, LR-CC at the lowest order includes single and double excitations (LR-CCSD), and scales as *O*(*n*^6^), where *n* is a measure of the system size (*i*.*e*., the number of orbitals). This computational cost keeps increasing as higher order excitations are included, and scales as *O*(*n*^8^) with the inclusion of triple excitations (LR-CCSDT) and *O*(*n*^10^) with the further inclusion of quadruple excitations (LR-CCSDTQ). As a result of this steep rise in the cost, such calculations are computationally prohibitive, even when one is dealing with relatively small molecules containing only 10–15 heavy (non-hydrogen) atoms. In addition to the computational cost required for a wavefunction-based treatment of the electron correlation, the error introduced by the use of a finite one-electron basis set is another factor that needs to be considered when computing ***α***. In this regard, basis set incompleteness error in the prediction of ***α*** can be more severe than the error due to the lack of higher order (*e*.*g*., beyond doubles) excitations^16–19^.

**Fig. 1:**
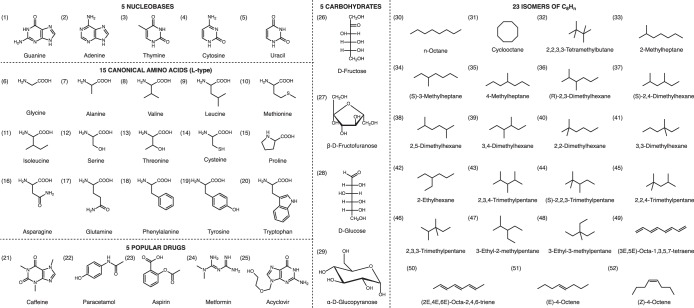
Names and indices of the 52 molecules in the AlphaML showcase database. (1–5) DNA/RNA nucleobases; (6–20) uncharged canonical amino acids (*L*-type); (21–25) popular pharmaceutical molecules; (26–29) open-chain and cyclic carbohydrates; and (30–52) 23 isomers of C_8_H_n_. Throughout this work, these molecules will be specified by “showcase” followed by the corresponding index (padded to four digits with leading zeros, *e*.*g*., showcase0001 to showcase0052).

In this work, we provide static (frequency-independent) ***α*** tensors computed using LR-CCSD and hybrid density functional theory (DFT) for all molecules in the QM7b^20–22^ and AlphaML showcase databases^23^. The QM7b database^20–22^ has become one of the *de facto* standard databases for machine-learning (ML) applications in chemistry, and contains *N* = 7,211 small organic molecules with up to seven heavy atoms (*i*.*e*., C, N, O, S, and Cl) and varying levels of H saturation. Recently introduced by Wilkins *et al*.^23^ for testing the transferability of ML-based predictions of ***α***, the AlphaML showcase database consists of *N* = 52 larger organic molecules (with up to 16 heavy atoms), and includes the DNA/RNA nucleobases, uncharged amino acids, several open-chain and cyclic carbohydrates, five popular pharmaceutical molecules, and 23 isomers of C_8_H_n_ (see Fig. 1). The diversity of structures in this combination of databases includes alkanes, alkenes, alkynes, (hetero)cycles, carbonyl and carboxyl groups, cyanides, amides, alcohols, amines, thiols, ethers, and epoxides, thereby providing a meaningful survey of ***α*** across a wide swath of chemical compound space.

Reference values for ***α*** were obtained with LR-CCSD with the doubly-augmented d-aug-cc-pVDZ basis set of Woon and Dunning^19^, as this method (when employed in conjunction with a sufficiently large and diffuse one-particle basis set) has been shown to yield accurate and reliable predictions for ***α***^16–18,24^. The use of d-aug-cc-pVDZ greatly mitigates the basis set incompleteness error at the double-*ζ* level, and the validity of this basis set choice will be critically examined and discussed in more detail below. For comparative purposes, we also provide finite-field DFT values for ***α*** obtained with the popular B3LYP^25,26^ and SCAN0^27^ hybrid functionals in conjunction with the d-aug-cc-pVDZ (B3LYP and SCAN0) and d-aug-cc-pVTZ (B3LYP only) basis sets. Throughout the remainder of this work, the d-aug-cc-pVXZ basis sets (with X = D and T) will be referred to as daXZ, and all LR-CCSD/daDZ calculations will simply be denoted by CCSD/daDZ unless otherwise specified.

## Methods

In this section, we provide the conventions used in generating and processing the geometries of the molecules in the QM7b and AlphaML showcase databases, all relevant computational details to ensure reproducibility of the quantum mechanical data, as well as a summary of the codes employed in this work.

### Molecular cartesian coordinates in the QM7b and AlphaML showcase databases

The molecular geometries for all 7,211 species in the QM7b database^20–22^ were obtained online *via* the quantum-machine.org website^28^. All QM7b molecular geometries were first translated to their respective center of nuclear (ionic) charge, to remove the origin-dependence of the higher-order (*i*.*e*., quadrupole) multipole moments. Using farthest-point sampling (FPS)^29^, all molecules were then reordered using a kernel-based similarity measure^30^, and relabelled accordingly from molecule0001 to molecule7211 (again padded to four digits with leading zeros). For consistency with the QM7b database, all 52 molecules in the AlphaML showcase database (see Fig. 1) were optimized with DFT using the PBE functional^31^ and a converged numerical atom-centered basis (*i*.*e*., tight settings with the tier-2 basis set in FHI-AIMS)^32^. All AlphaML showcase molecules were also translated to their respective center of nuclear (ionic) charge, and are labelled from showcase0001 to showcase0052, as depicted in Fig. 1. All 7,263 structures are available on Materials Cloud^33^, according to the format described below in the *Data Records* section.

### Details of the quantum mechanical calculations

All CCSD/daDZ, B3LYP/daDZ, and B3LYP/daTZ calculations were carried out using Psi4 v1.1^34^, while all SCAN0/daDZ calculations were performed with Q-Chem v5.0^35^. At the CCSD/daDZ level, all ***α*** tensors, unrelaxed dipole moments, ***μ***, and unrelaxed quadrupole moments, ***Q***, were calculated using LR-CCSD/daDZ, with the exception of the ten largest molecules in the AlphaML showcase database (*e*.*g*., (18) Phenylalanine, (19) Tyrosine, (20) Tryptophan, (21) Caffeine, (23) Aspirin, (25) Acyclovir, (26) D-Fructose, (27) *β*-D-Fructofuranose, (28) D-Glucose, and (29) *α*-D-glucopyranose, see Fig. 1). For these molecules, the memory requirements required to solve the Λ-CC equations at the LR-CCSD/daDZ level were computationally prohibitive, and only energy calculations with CCSD/daDZ could be performed with the available computational resources. For consistency, this required the use of the orbital-*unrelaxed* finite-field method, in which the molecular orbitals were obtained from a field-free (unperturbed) Hartree-Fock calculation. To obtain ***μ*** and ***α***, we computed first and second derivatives of the CCSD/daDZ energy (*U*) with respect to an external electric field, **E**, *i*.*e*., ***μ*** = ∂*U*/∂**E** and ***α*** = ∂^2^*U*/∂**E**^2^. ***Q*** values were not computed for the ten largest molecules in the AlphaML showcase database. All DFT calculations used the orbital-*relaxed* finite-field method, in which a self-consistent field (SCF) was obtained in the presence of each applied field, and ***α*** was computed via ***α*** = ∂***μ***/∂**E**. All other molecular properties at the DFT level (*vide infra*) were obtained directly from the field-free (unperturbed) calculation. All derivatives were computed numerically using two-point (for first derivatives) and three-point (for second derivatives) central difference formulae and a step size of **E** = 1.8897261250 × 10^−5^ atomic units.

For all LR-CCSD/daDZ calculations, the convergence criteria were set to their default values in Psi4, *i*.*e*., E_convergence = 1.0E-10 and D_convergence = 1.0E-10 for the energy and density during the solution of the HF equations, and E_convergence = 1.0E-08 and R_convergence = 1.0E-07 for the energy and residuals during the solution of the CCSD equations. For the ten largest molecules in the AlphaML showcase database, the finite-field CCSD/daDZ calculations were performed using the following convergence criteria in Psi4: E_convergence = 5.0E-10 and D_convergence = 5.0E-10 for the energy and density during the solution of the HF equations. Significantly tighter convergence criteria of E_convergence = 5.0E-10 and R_convergence = 5.0E-09 were employed for the energy and residuals during the solution of the CCSD equations to minimize errors in the numerical evaluation of ***μ*** and ***α***. The frozen core (FC) approximation and scf_type = direct were used for all LR-CCSD/daDZ and CCSD/daDZ calculations. For all B3LYP/daDZ and B3LYP/daTZ calculations, the convergence criteria in Psi4 were again set to tight values to minimize numerical error in the finite-difference evaluation of ***α***: E_convergence = 1.0E-10 and D_convergence = 1.0E-10 for the energy and density during the solution of the Kohn-Sham equations. For all the SCAN0/daDZ calculations, the convergence criteria were set to scf_convergence = 1.0E-10 and thresh = 1.0E-13 for the DIIS error and integral thresholding in Q-Chem. The Dunning-style daDZ and daTZ basis sets^19^ were obtained from the EMSL Basis Set Library^36,37^.

## Data Records

In this section, we briefly describe the molecular properties that have been computed in this work, as well as the conventions used to store and retrieve the generated data. In addition to a select set of molecular properties (such as energetic components, dipole and quadrupole moments, orbital eigenvalues, etc.), the provided data will also include the full output files from all of the calculations performed herein. In what follows, we focus the discussion on ***α***, as this molecular response property is arguably the most challenging quantity computed in this work. In particular, we provide a statistical summary of the CCSD/daDZ ***α*** data in the QM7b and AlphaML databases, as well as a comparative analysis of the different quantum mechanical methods employed in this work.

### Included molecular properties and file format

To store and disseminate the data generated in this work, we have created the following four data packages: CCSD_daDZ, B3LYP_daDZ, SCAN0_daDZ, and B3LYP_daTZ. Each data package contains 7,263 standard xyz files, and has been named according to the level of theory used to generate the data contained therein. Each of the included xyz files contains the translated geometries and calculated properties for a single molecule in the QM7b and AlphaML showcase databases. As described above, the 7,211 molecules in the QM7b database are contained in xyz files labelled from molecule0001 to molecule7211, and the 52 molecules in the AlphaML showcase database are contained in xyz files labelled from showcase0001 to showcase0052 (see Fig. 1).

All computed properties (for a given molecule) are provided on the “comment line” (*i*.*e*., the second line) of the corresponding xyz file (as comma-separated values), following the order provided in Table 1 (for CCSD_daDZ) and Table 2 (for B3LYP_daDZ, SCAN0_daDZ, and B3LYP_daTZ). Common molecular properties included in all four data packages are: the isotropic polarizability (*α*_iso_),

1 $${\alpha }_{{\rm{iso}}}=\frac{1}{3}({\alpha }_{{\rm{xx}}}+{\alpha }_{{\rm{yy}}}+{\alpha }_{{\rm{zz}}}),$$

anisotropic polarizability (*α*_aniso_),

2 $$\begin{array}{lll}{\alpha }_{{\rm{aniso}}} & = & \frac{1}{\sqrt{2}}\left[{({\alpha }_{{\rm{xx}}}-{\alpha }_{{\rm{yy}}})}^{2}+{({\alpha }_{{\rm{yy}}}-{\alpha }_{{\rm{zz}}})}^{2}\right.\\  &  & {\left.+{({\alpha }_{{\rm{zz}}}-{\alpha }_{{\rm{xx}}})}^{2}+6({\alpha }_{{\rm{xy}}}^{2}+{\alpha }_{{\rm{xz}}}^{2}+{\alpha }_{{\rm{yz}}}^{2})\right]}^{1/2},\end{array}$$

all symmetry-unique components of the polarizability (***α***) tensor (*i*.*e*., *α*_xx_, *α*_yy_, *α*_zz_, *α*_xy_, *α*_xz_, *α*_yz_), all components of the dipole moment (***μ***) vector (*i*.*e*., *μ*_x_, *μ*_y_, *μ*_z_), and all symmetry-unique components of the quadrupole moment (***Q***) vector (*i*.*e*., *Q*_xx_, *Q*_yy_, *Q*_zz_, *Q*_xy_, *Q*_xz_, *Q*_yz_). In the CCSD_daDZ data package only, the following molecular properties are also included: the Hartree-Fock total energy (${E}_{{\rm{tot}}}^{{\rm{HF}}}$), same-spin (${E}_{{\rm{ss}}}^{{\rm{MP}}2}$) and opposite-spin (${E}_{{\rm{os}}}^{{\rm{MP}}2}$) correlation energies at the level of second-order Møller-Plesset perturbation (MP2) theory, and same-spin (${E}_{{\rm{ss}}}^{{\rm{CCSD}}}$) and opposite-spin (${E}_{{\rm{os}}}^{{\rm{CCSD}}}$) correlation energies at the CCSD level. In the B3LYP_daDZ, SCAN0_daDZ, and B3LYP_daTZ data packages only, the following molecular properties are also included: the DFT total energy (${E}_{{\rm{tot}}}^{{\rm{DFT}}}$) and the eigenvalues corresponding to the HOMO (${\epsilon }_{{\rm{HOMO}}}$) and LUMO (${\epsilon }_{{\rm{LUMO}}}$). All data described above is provided in atomic units and available for download on Materials Cloud^33^.

**Table 1:** Calculated properties at the CCSD/daDZ level.

No.	Property	Description
01	Tag	“Properties” string
02	*α*_iso_	isotropic polarizability (see Eq. (1))
03	*α*_aniso_	anisotropic polarizability (see Eq. (2))
04–09	***α***	polarizability tensor^*a*^
10–12	***μ***	(unrelaxed) dipole moment^*b*^
13–18	***Q***	(unrelaxed) quadrupole moment^*c*,*d*^
19	${E}_{{\rm{tot}}}^{{\rm{HF}}}$	HF total energy
20	${E}_{{\rm{ss}}}^{{\rm{MP}}2}$	MP2 same-spin correlation energy
21	${E}_{{\rm{os}}}^{{\rm{MP}}2}$	MP2 opposite-spin correlation energy
22	${E}_{{\rm{ss}}}^{{\rm{CCSD}}}$	CCSD same-spin correlation energy
23	${E}_{{\rm{os}}}^{{\rm{CCSD}}}$	CCSD opposite-spin correlation energy

All properties are in atomic units and are provided on the “comment line” (*i*.*e*., the second line) of a standard xyz file.

^*a*^04–09: *α*_xx_, *α*_yy_, *α*_zz_, *α*_xy_, *α*_xz_, *α*_yz_.

^*b*^10–12: *μ*_x_, *μ*_y_, *μ*_z_.

^c^13–18: *Q*_xx_, *Q*_yy_, *Q*_zz_, *Q*_xy_, *Q*_xz_, *Q*_yz_.

^*d*^***Q*** values are not provided for the ten largest molecules in the AlphaML showcase database (see text for details).

**Table 2:** Calculated properties at the B3LYP/daDZ, SCAN0/daDZ, and B3LYP/daTZ levels.

No.	Properties	Description
01	Tag	“Properties” string
02	*α*_iso_	isotropic polarizability (see Eq. (1))
03	*α*_aniso_	anisotropic polarizability (see Eq. (2))
04–09	***α***	polarizability tensor^*a*^
10–12	***μ***	dipole moment^*b*^
13–18	***Q***	quadrupole moment^*c*^
19	${E}_{{\rm{tot}}}^{{\rm{DFT}}}$	DFT total energy
20	${\epsilon }_{{\rm{HOMO}}}$	HOMO energy
21	${\epsilon }_{{\rm{LUMO}}}$	LUMO energy

All properties are in atomic units and are provided on the “comment line” (*i*.*e*., the second line) of a standard xyz file.

^*a*^04–09: *α*_xx_, *α*_yy_, *α*_zz_, *α*_xy_, *α*_xz_, *α*_yz_.

^*b*^10–12: *μ*_x_, *μ*_y_, *μ*_z_.

^*c*^13–18: *Q*_xx_, *Q*_yy_, *Q*_zz_, *Q*_xy_, *Q*_xz_, *Q*_yz_.

### Statistical summary of the CCSD/daDZ *α* data

**Fig. 2:**
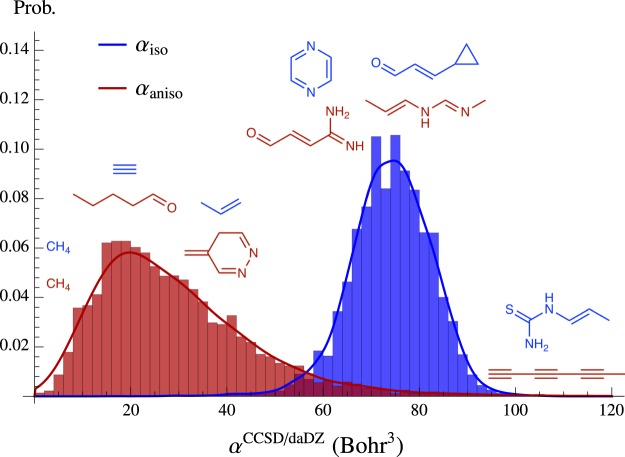
Normalized probability distributions of the CCSD/daDZ isotropic (*α*_iso_, blue) and anisotropic (*α*_aniso_, red) polarizabilities in the QM7b database. Molecules with the smallest and largest *α*_iso_ and *α*_aniso_ values are depicted at the left and right of the figure, respectively. Molecules with intermediate *α*_iso_ and *α*_aniso_ values (≈20, 40, 60, and 80 Bohr^3^) are depicted in the middle of the figure. Histogram bin widths were set to 2.0 Bohr^3^.

To provide an overview of the ***α*** data, Fig. 2 contains the normalized probability distributions of the CCSD/daDZ isotropic (*α*_iso_, blue) and anisotropic (*α*_aniso_, red) polarizabilities in the QM7b database. This is accompanied by Table 3, which provides a statistical analysis of all ***α*** data generated in this work. From Fig. 2 and Table 3, one can see that the CCSD/daDZ *α*_iso_ values in the QM7b database have a range of 16.80–106.50 Bohr^3^, and are centered around a mean value of 〈*α*〉 = 74.07 Bohr^3^. With a standard deviation (*σ*) that is nearly two times larger than *α*_iso_, the CCSD/daDZ *α*_aniso_ values in this database are characterized by a broader distribution that is significantly skewed to the right. We note in passing that the range of *α*_aniso_ is larger than *α*_iso_ by ≈64%, and includes minimum values that are significantly smaller (*cf*. ${\alpha }_{{\rm{aniso}}}^{{\rm{\min }}}=2.16\times 1{0}^{-4}$ Bohr^3^ vs. ${\alpha }_{{\rm{iso}}}^{{\rm{\min }}}=16.80$ Bohr^3^) and maximum values that are significantly larger (*cf*. ${\alpha }_{{\rm{aniso}}}^{{\rm{\max }}}=147.49$ Bohr^3^ vs. ${\alpha }_{{\rm{iso}}}^{{\rm{\max }}}=106.50$ Bohr^3^). Also depicted in Fig. 2 are the subset of molecules in the QM7b database with the smallest and largest *α*_iso_ and *α*_aniso_ values (as well as those molecules with intermediate *α*_iso_ and *α*_aniso_ values of ≈20, 40, 60, and 80 Bohr^3^). From these molecules, one clearly sees that *α*_iso_ is an extensive quantity that grows with molecular size, and *α*_aniso_ (which is a measure of the anisotropy in the ***α*** tensor, see Eq. (2)) is largest for molecules with elongated and non-spherical/asymmetric shapes.

**Table 3:** Statistical analysis of the isotropic (*α*_iso_) and anisotropic (*α*_aniso_) polarizabilities in the QM7b and AlphaML showcase databases computed at the CCSD/daDZ, B3LYP/daDZ, SCAN0/daDZ, and B3LYP/daTZ levels.

*α*	Level	〈*α*〉	*σ*	*α* ^min^	*α* ^max^
**QM7b Database**					
*α*_iso_	CCSD/daDZ	74.07	8.47	16.80	106.50
	B3LYP/daDZ	75.97	9.14	17.32	117.29
	SCAN0/daDZ	74.27	9.06	16.94	116.64
	B3LYP/daTZ	75.90	9.14	17.30	117.27
*α*_aniso_	CCSD/daDZ	29.09	16.18	2.16E-4	147.49
	B3LYP/daDZ	32.08	18.93	4.13E-4	181.51
	SCAN0/daDZ	31.51	18.58	3.83E-3	177.21
	B3LYP/daTZ	32.08	18.94	2.74E-4	181.74
**AlphaML Showcase Database**					
*α*_iso_	CCSD/daDZ	99.59	20.87	43.94	156.44
	B3LYP/daDZ	101.56	22.14	44.33	156.38
	SCAN0/daDZ	99.37	21.97	42.75	153.08
	B3LYP/daTZ	101.42	22.14	44.25	156.24
*α*_aniso_	CCSD/daDZ	35.66	32.52	6.93	171.44
	B3LYP/daDZ	39.19	40.07	7.52	229.32
	SCAN0/daDZ	38.35	39.91	7.36	231.07
	B3LYP/daTZ	39.18	40.11	7.56	229.66

Statistical quantities (in Bohr^3^) include: 〈*α*〉 (mean), *σ* (standard deviation), *α*^min^ (minimum value), and *α*^max^ (maximum value).

The statistical summary of the ***α*** data corresponding to the 52 molecules in the AlphaML showcase database (see Table 3) also illustrates that the *α*_iso_ (*α*_aniso_) distributions in this database are characterized by 〈*α*〉 values that are larger by ≈34% (≈23%) and *σ* values that are 2.5× (2.0×) larger than that found in the QM7b database. In addition, the range of *α*_iso_ (*α*_aniso_) values is approximately 25% (12%) larger in the AlphaML showcase database, and does not include symmetric molecules with vanishingly small *α*_aniso_ values. Taken together, these statistical measures reflect the fact that the molecules in the AlphaML showcase database, which includes the DNA/RNA nucleobases, uncharged amino acids, several open-chain and cyclic carbohydrates, five popular pharmaceutical molecules, and 23 isomers of C_8_H_n_, are (in general) larger and more diverse than those contained in the QM7b database (see Fig. 1).

### Comparative analysis of the quantum mechanical methodologies

**Fig. 3:**
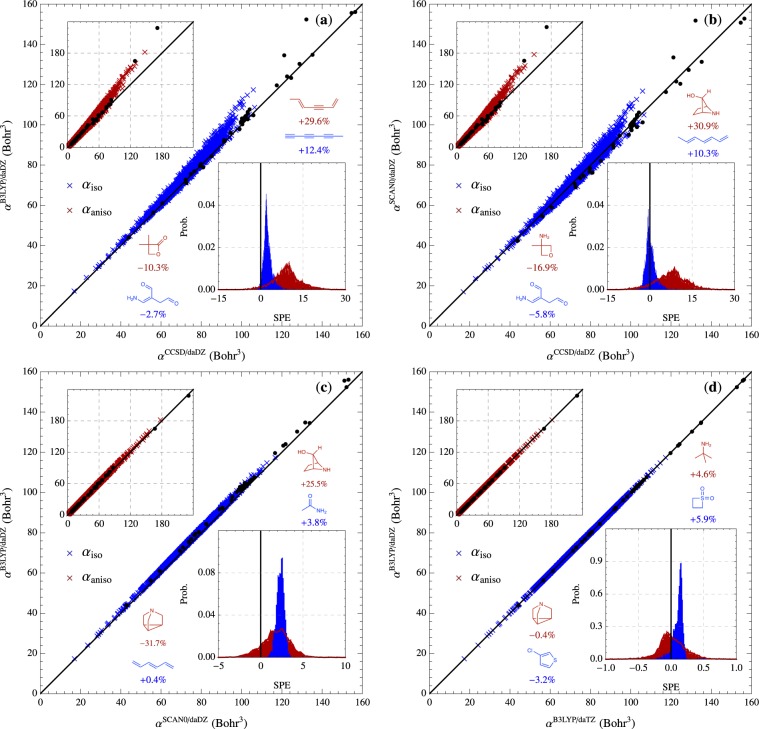
Correlation plots of the isotropic (*α*_iso_, blue, main plots) and anisotropic (*α*_aniso_, red, upper left insets) polarizabilities in the QM7b and AlphaML showcase databases computed at different levels of theory. Normalized probability distributions of the signed percent errors (SPE, computed with respect to the Reference Level of theory on the *x*-axis) are provided in the lower right insets of each panel. Histogram bin widths were set to 0.1% for panels (a) B3LYP/daDZ vs. CCSD/daDZ, (b) SCAN0/daDZ vs. CCSD/daDZ, and (c) B3LYP/daDZ vs. SCAN0/daDZ, and 0.01% for panel (d) B3LYP/daDZ vs. B3LYP/daTZ. For completeness, *α*_iso_ and *α*_aniso_ values in the AlphaML showcase database are depicted as black circles in the main plots and upper left insets of each panel. Molecules corresponding to the minimum and maximum *α*_iso_ and *α*_aniso_ SPE values are also depicted in each panel. When computing the minimum and maximum *α*_aniso_ SPE values, molecules with very small *α*_aniso_ values (*i*.*e*., molecule0001: methane, ${\alpha }_{{\rm{aniso}}}^{{\rm{CCSD/daDZ}}}=2.16\times 1{0}^{-4}$ Bohr^3^ and molecule1009: neopentane, ${\alpha }_{{\rm{aniso}}}^{{\rm{CCSD/daDZ}}}=3.83\times 1{0}^{-4}$ Bohr^3^) were excluded, as these molecules yielded large (but physically insignificant) SPE values.

To investigate the performance of different quantum mechanical methodologies in calculating the ***α*** tensor in the QM7b and AlphaML showcase databases, a detailed statistical error analysis was carried out for the following combinations of methods (Level//Reference Level): B3LYP/daDZ//CCSD/daDZ and SCAN0/daDZ//CCSD/daDZ (to compare the electron correlation level while keeping the basis set fixed), B3LYP/daDZ//SCAN0/daDZ (to compare the exchange-correlation functional while keeping the basis set fixed), and B3LYP/daDZ//B3LYP/daTZ (to quantify the basis set incompleteness error at the B3LYP level). A summary of statistical error measures, including the mean signed error, ${\rm{MSE}}\equiv \frac{1}{N}{\sum }_{i=1}^{N}({\alpha }_{i}-{\alpha }_{i}^{{\rm{ref}}})$, mean absolute error, ${\rm{MAE}}\equiv \frac{1}{N}{\sum }_{i=1}^{N}\left|{\alpha }_{i}-{\alpha }_{i}^{{\rm{ref}}}\right|$, and root-mean-square error, ${\rm{RMSE}}\equiv \sqrt{\frac{1}{N}{\sum }_{i=1}^{N}{({\alpha }_{i}-{\alpha }_{i}^{{\rm{ref}}})}^{2}}$, are provided in Table 4 as well as the corresponding percent errors, ${\rm{M}}{\rm{S}}{\rm{P}}{\rm{E}}\equiv \frac{1}{N}{\sum }_{i=1}^{N}(\frac{{\alpha }_{i}-{\alpha }_{i}^{{\rm{r}}{\rm{e}}{\rm{f}}}}{{\alpha }_{i}^{{\rm{r}}{\rm{e}}{\rm{f}}}})\times 100{\rm{ \% }}$, ${\rm{MAPE}}\equiv \frac{1}{N}\mathop{\sum }\limits_{i=1}^{N}\,\left|\frac{{\alpha }_{i}-{\alpha }_{i}^{{\rm{ref}}}}{{\alpha }_{i}^{{\rm{ref}}}}\right|\times 100{\rm{ \% }}$, and ${\rm{RMSPE}}\equiv \sqrt{\frac{1}{N}\mathop{\sum }\limits_{i=1}^{N}\,{\left(\frac{{\alpha }_{i}-{\alpha }_{i}^{{\rm{ref}}}}{{\alpha }_{i}^{{\rm{ref}}}}\right)}^{2}}\times 100{\rm{ \% }}$. To visualize these differences in more detail, correlation plots (corresponding to the four method combinations above) for *α*_iso_ and *α*_aniso_ (as well as probability distributions of the signed percent error (SPE)) are provided in Fig. 3.

**Table 4:** Comparative analysis of the isotropic (*α*_iso_) and anisotropic (*α*_aniso_) polarizabilities in the QM7b and AlphaML showcase databases computed at the CCSD/daDZ, B3LYP/daDZ, SCAN0/daDZ, and B3LYP/daTZ levels.

*α*	Level	Reference Level	MSE (MSPE)	MAE (MAPE)	RMSE (RMSPE)
**QM7b Database**					
*α*_iso_	B3LYP/daDZ	CCSD/daDZ	1.91 (2.52)	1.92 (2.54)	2.32 (2.97)
	SCAN0/daDZ	CCSD/daDZ	0.20 (0.21)	0.97 (1.29)	1.41 (1.78)
	B3LYP/daDZ	SCAN0/daDZ	1.70 (2.31)	1.70 (2.31)	1.73 (2.36)
	B3LYP/daDZ	B3LYP/daTZ	0.08 (0.11)	0.09 (0.12)	0.11 (0.14)
*α*_aniso_	B3LYP/daDZ	CCSD/daDZ	2.99 (9.19)	3.03 (9.34)	4.48 (10.4)
	SCAN0/daDZ	CCSD/daDZ	2.42 (7.34)	2.59 (7.93)	4.01 (9.25)
	B3LYP/daDZ	SCAN0/daDZ	0.57 (1.78)	0.64 (2.09)	0.85 (2.52)
	B3LYP/daDZ	B3LYP/daTZ	<0.01 (0.02)	0.05 (0.17)	0.07 (0.25)
**AlphaML Showcase Database**					
*α*_iso_	B3LYP/daDZ	CCSD/daDZ	1.97 (1.83)	2.21 (2.09)	3.87 (3.22)
	SCAN0/daDZ	CCSD/daDZ	−0.22 (−0.43)	2.15 (2.08)	3.81 (3.24)
	B3LYP/daDZ	SCAN0/daDZ	2.19 (2.29)	2.19 (2.29)	2.34 (2.44)
	B3LYP/daDZ	B3LYP/daTZ	0.13 (0.14)	0.13 (0.14)	0.14 (0.15)
*α*_aniso_	B3LYP/daDZ	CCSD/daDZ	3.52 (7.83)	3.66 (8.10)	9.87 (9.97)
	SCAN0/daDZ	CCSD/daDZ	2.69 (5.25)	3.37 (6.73)	10.0 (9.11)
	B3LYP/daDZ	SCAN0/daDZ	0.84 (2.57)	0.94 (2.65)	1.44 (3.57)
	B3LYP/daDZ	B3LYP/daTZ	<0.01 (0.01)	0.08 (0.23)	0.11 (0.31)

Statistical quantities (in Bohr^3^) are computed with respect to the Reference Level and include: mean signed error (MSE), mean absolute error (MAE), and root-mean-square error (RMSE). Corresponding percent errors (MSPE, MAPE, RMSPE) are provided in %. When computing the MSPE, MAPE, and RMSPE values for *α*_aniso_ in the QM7b database, molecules with very small *α*_aniso_ values (*i*.*e*., molecule0001: methane, ${\alpha }_{{\rm{aniso}}}^{{\rm{CCSD/daDZ}}}=2.16\times 1{0}^{-4}$ Bohr^3^ and molecule1009: neopentane, ${\alpha }_{{\rm{aniso}}}^{{\rm{CCSD/daDZ}}}=3.83\times 1{0}^{-4}$ Bohr^3^) were excluded, as these molecules yielded large (but physically insignificant) percent errors.

When comparing B3LYP/daDZ to the reference CCSD/daDZ level for the molecules in the QM7b database, one sees that B3LYP/daDZ yields essentially identical MSE and MAE values for *α*_iso_ (*i*.*e*., 1.91 Bohr^3^ and 1.92 Bohr^3^, respectively), indicating that B3LYP/daDZ systematically overestimates *α*_iso_ values by ≈2.5% (see Table 4). With an RMSE value that is ≈21% greater than the MAE, the magnitudes of the B3LYP/daDZ errors show substantial variations from molecule to molecule; this is particularly evident for the molecules with large *α*_iso_ values in Fig. 3(a). When comparing SCAN0/daDZ to CCSD/daDZ, one sees that SCAN0/daDZ outperforms B3LYP/daDZ by a large margin in the prediction of *α*_iso_, yielding reductions of ≈90%, ≈50%, and ≈40% in the MSE, MAE, and RMSE values, respectively. In this regard, our finding that the SCAN0 functional provides greatly improved estimates for *α*_iso_ is also consistent with the recent benchmark study by Lao *et al*.^18^ on the dipole polarizability surface of the gas-phase water molecule. With an MSE value that is nearly 5× smaller than the MAE, it is also worth noting that SCAN0/daDZ *α*_iso_ values only have a slightly positive systematic error. From a quick glance at Fig. 3(b), it is clear that SCAN0/daDZ (like B3LYP/daDZ) also has more difficulties when treating molecules with large *α*_iso_ values; this is indicative of the challenges that one faces when computing ***α***, a response property which becomes substantially more non-additive as the size and complexity of the molecules increase. When comparing B3LYP/daDZ to SCAN0/daDZ, one obtains nearly identical MSE, MAE, and RMSE values, which indicates that: (*i*) B3LYP/daDZ systematically overestimates *α*_iso_ with respect to SCAN0/daDZ, and (*ii*) the magnitudes of the B3LYP/daDZ errors do not show substantial molecule-to-molecule variations. Both of these findings are confirmed in Fig. 3(c), where one sees that: (*i*) the SPE distribution is centered around 2.3% (and not zero), and (*ii*) there is clearly a very strong linear correlation between the B3LYP/daDZ and SCAN0/daDZ *α*_iso_ values that does not deteriorate with molecular size and complexity. When comparing B3LYP/daDZ to B3LYP/daTZ, one finds that the increase from double- to triple-*ζ* in the underlying basis set does not lead to significantly different *α*_iso_ values. This finding is consistent with the (in general) rapid convergence of DFT with respect to the occupied space and the relatively weak dependence of DFT on the virtual/unoccupied space.

From a quick glance at Table 4, one also sees that both B3LYP and SCAN0 (when compared to the reference CCSD/daDZ level) yield larger errors when predicting *α*_aniso_ than *α*_iso_. When comparing B3LYP/daDZ to CCSD/daDZ, for example, the MSPE, MAPE, and RMSPE values increased from 2.52%, 2.54%, and 2.97% for *α*_iso_ to 9.19%, 9.34%, and 10.4% for *α*_aniso_; a similar increase was observed when comparing SCAN0/daDZ to CCSD/daDZ. These findings demonstrate that the tensorial properties of ***α*** (which govern *α*_aniso_) are more difficult to predict than the average of the diagonal elements (*i*.*e*., *α*_iso_ values). When compared to CCSD/daDZ, SCAN0/daDZ is no longer performing substantially better than B3LYP/daDZ and now exhibits a (nearly) systematic overestimation of *α*_aniso_. By looking at the upper left insets in Fig. 3(a,b), one again sees that the errors made by B3LYP and SCAN0 increase for molecules with larger *α*_aniso_ values; this is indicative of the increasing importance of including electron correlation effects when predicting *α*_aniso_ for molecules that are larger in size and potentially more anisotropic. Among the DFT functionals at the daDZ level, one sees that B3LYP/daDZ overestimates *α*_aniso_ with respect to SCAN0/daDZ in most cases, and that B3LYP/daDZ and SCAN0/daDZ are now in better agreement with each other than with CCSD/daDZ. When comparing B3LYP/daDZ and B3LYP/daTZ, the B3LYP functional again shows rapid convergence with respect to the underlying basis set in the prediction of *α*_aniso_.

When performing a similar analysis for the molecules in the AlphaML showcase database, most of the findings described above for the QM7b database still hold. One interesting distinction is the finding that SCAN0/daDZ no longer outperforms B3LYP/daDZ when predicting *α*_iso_ values for the larger and more complex molecules contained in the AlphaML showcase database; in the same breath, we note that SCAN0/daDZ still maintains a relatively small MSE value, which is indicative of an error profile that is more random (and less systematic) than B3LYP/daDZ (see Fig. 3(a,b)).

## Technical Validation

In this section, we explore the validity and reliability of the CCSD/daDZ ***α*** data in the QM7b database.

### Validation of the CCSD/daDZ *α* Data

Since ***α*** describes the response of a molecule to an applied electric field, an accurate and reliable treatment of this quantity is particularly sensitive to the description of the underlying electronic structure as well as the quality of the basis set. The highest level ***α*** values provided in this work were computed with LR-CCSD, a sophisticated wavefunction-based method that consistently yields highly accurate ***α*** values for equilibrium and non-equilibrium molecular geometries when used with sufficiently large (and sufficiently diffuse) basis sets^16–18,24^. To account for the basis set incompleteness error, which is almost always larger than the contributions from higher-order (*e*.*g*., beyond doubles) excitations in coupled-cluster theory^16–19^, we employed the daDZ basis set. Although daDZ is a double-*ζ* basis set containing a moderate number of polarization functions, the incorporation of two sets of augmented functions (*i*.*e*., double augmentation) significantly reduces the basis set incompleteness error in the prediction of ***α***. To validate the accuracy of our CCSD/daDZ calculations, we performed a series of calculations using the larger daTZ basis set^19^, which is arguably the largest Dunning-style basis that can be used to compute ***α*** for the molecules in the QM7b database without significant supercomputer resources. To proceed with this technical validation, we used the FPS algorithm^29,30^ to choose the 100 most diverse molecules in the QM7b database (which we denote as the FPS-100 database). Due to the prohibitively large computational cost associated with LR-CCSD calculations with the daTZ basis set, we were only able to compute ***α*** for the 24 smallest molecules (by number of basis functions) in the FPS-100 database. A statistical error analysis of the *α*_iso_ and *α*_aniso_ values for these 24 molecules is provided in Table 5, and a more extensive discussion regarding the basis set convergence of our CCSD/daDZ calculations can be found in the main text and Supplementary Information of Ref.^23^. From Table 5, one can immediately see that the CCSD/daDZ *α*_iso_ values have similar MSE, MAE, and RMSE values of ≈0.20 Bohr^3^, which corresponds to a MAPE of ≈0.4%. For *α*_aniso_, a measure of the anisotropy in the ***α*** tensor, we report slightly larger errors corresponding to a MAPE of $\lesssim 1{\rm{ \% }}$. When compared to the errors made by the hybrid DFT functionals employed in this work (with CCSD/daDZ as the reference), namely 2.5% (B3LYP/daDZ) and 1.3% (SCAN0/daDZ) for *α*_iso_ and 9.3% (B3LYP/daDZ) and 7.9% (SCAN0/daDZ) for *α*_aniso_, we conclude that the basis set incompleteness errors in our reference ***α*** values are significantly smaller (see Table 4). As such, the CCSD/daDZ ***α*** tensors presented in this work should be accurate and reliable enough for use in the development (and assessment) of next-generation force fields, density functionals, and quantum chemical methodologies, as well as machine-learning based approaches for predicting this fundamental response property.

**Table 5:** Statistical error analysis of the CCSD/daDZ isotropic (*α*_iso_) and anisotropic (*α*_aniso_) polarizabilities in the FPS-100 database (*i*.*e*., the first 100 molecules in the QM7b database chosen by the FPS algorithm) computed with respect to the CCSD/daTZ level.

*α*	MSE (MSPE)	MAE (MAPE)	RMSE (RMSPE)
*α*_iso_	0.22 (0.42)	0.22 (0.43)	0.26 (0.50)
*α*_aniso_	−0.25 (−0.79)	0.27 (0.87)	0.33 (1.15)

Due to the increased computational cost associated with computing ***α*** at the CCSD/daTZ level, a subset of the FPS-100 database (which includes the 24 molecules with the smallest number of basis functions) was considered during this analysis.

## ISA-Tab metadata file

Download metadata file

## Data Availability

As mentioned above, three different software packages were utilized in this work. Psi4 v1.1^34^ is freely available from its official website^38^ Q-Chem v5.0^35^ and FHI-AIMS^32^ must be downloaded from their official sites^39,40^ with a signed license.
